# Detection of Parietaria Mottle Virus by RT-qPCR: An Emerging Virus Native of Mediterranean Area That Undermine Tomato and Pepper Production in Southern Italy

**DOI:** 10.3389/fpls.2021.698573

**Published:** 2021-09-03

**Authors:** Stefano Panno, Andrea Giovanni Caruso, Sofia Bertacca, Slavica Matić, Salvatore Davino, Giuseppe Parrella

**Affiliations:** ^1^Department of Agricultural, Food and Forest Sciences, University of Palermo, Palermo, Italy; ^2^Department of Biological, Chemical and Pharmaceutical Science and Technologies, University of Palermo, Palermo, Italy; ^3^Institute for Sustainable Plant Protection, National Research Council (IPSP-CNR), Turin, Italy; ^4^Consorzio di Ricerca sul Rischio Biologico in Agricoltura (Co.Ri.Bi.A.), Palermo, Italy; ^5^Institute for Sustainable Plant Protection, National Research Council (IPSP-CNR), Portici, Italy

**Keywords:** PMoV, RT-qPCR, emerging virus, early detection, field survey

## Abstract

Parietaria mottle virus (PMoV) is considered an emerging virus in many countries of the Mediterranean basin, especially on tomato and pepper crops. Symptoms on tomato leaves and fruits can be easily confused with those induced by cucumber mosaic virus (CMV) with necrogenic satellite RNA (CMV-satRNA), tomato spotted wilt virus (TSWV) or tomato mosaic virus (ToMV). Mixed infection of these viruses has been also reported in some tomato cultivars, with an increase in the complexity of the symptoms and severity of the disease. Although a specific serum and riboprobes have been produced, nowadays no sensitive diagnostic methods are available for the rapid PMoV detection. Here, we have developed a RT-qPCR assay with the aim to establish a more sensitive and specific method for PMoV detection. Specific primers and TaqMan probe were designed and *in silico* tested with all PMoV isolates available in GenBank. Moreover, this method was evaluated on tomato naturally infected samples from Sicily region (Italy). Results obtained showed that the RT-qPCR assay developed in this work is extremely sensitive, in fact, it is able to detect as few as 10 PMoV RNA copies in tomato total RNA; moreover, it will be a particularly valuable tool for early detection of PMoV. Furthermore, the analyzes on field samples show how this pathogen is increasingly present in tomato crops in the last years, helping to undermine the Italian horticultural sector.

## Introduction

Vegetable crops represent one of the main sources of income and food worldwide. Among these crops of particular importance are tomatoes and peppers. Tomato (*Solanum lycopersicum* L., family *Solanaceae*) is a very important vegetable crop cultivated worldwide, with an area harvested of over five million hectares and a worldwide production of over 180 million tons (Food and Agriculture Organization of the United Nations (FAO), 2021). In the 2009–2019 decade, the global tomato production increased by more than 25 million tons (Food and Agriculture Organization of the United Nations (FAO), 2021). Another important horticultural crop is the pepper (*Capsicum annuum* L., family *Solanaceae*), that is cultivated worldwide in almost two million hectares, with a total production of 38 million tons.

In Italy, tomato production in open field is mainly located in Apulia and Emilia-Romagna regions, whereas tomato production in protected conditions is primarily based in Sicily (Agri ISTAT, 2020).

In the Mediterranean basin, Italy represents the main access point for plant material to European countries, due to its central geographical position; this significantly increases the danger of introducing new pathogens, posing a serious risk to agriculture, biosecurity, and food production, jeopardizing the future of Italian horticulture (Panno et al., 2019c).

In the last decades, emerging and re-emerging viral diseases caused significant losses in the horticultural sector (Hanssen et al., 2010). In particular, Italian horticultural production has faced significant losses, due to the appearance or recrudescence of several viral diseases, including tomato yellow leaf curl virus (TYLCV), tomato yellow leaf curl Sardinia virus (TYLCSV) (Davino et al., 2009, 2012) and their recombinants (Panno et al., 2018), tomato spotted wilt virus (TSWV) (Panno et al., 2012), pepino mosaic virus (PepMV) (Davino et al., 2017b), tomato leaf curl New Delhi virus (ToLCNDV) (Panno et al., 2019b), tomato brown rugose fruit virus (ToBRFV) (Panno et al., 2019a, 2020b), and most recently, parietaria mottle virus (PMoV) (Parrella et al., 2020).

PMoV, genus *Ilarvirus* (subgroup 1), family *Bromoviridae*, was identified and characterized for the first time in 1987 in Turin province (northern Italy) in *Parietaria officinalis*, causing a typical mottling in leaves (Caciagli et al., 1989) and subsequently in tomato (Lisa et al., 1998) and pepper (Janssen et al., 2005) plants. PMoV belongs to the same subgroup of tobacco streak virus (TSV), the type member of the genus *Ilarvirus*, but these two viruses are not serologically correlated.

PMoV genome is composed of three positive sense single-stranded RNAs (Galipienso et al., 2009): the RNA1 (4.3 kb) encoding a putative replicase (p1); the RNA2 (2.3 kb) encoding a putative replicase (p2) and a protein of unknown function (2b) (Shimura et al., 2013); the RNA3 (2.7 kb) encoding the movement protein (MP) and the coat protein (CP) that is expressed through the sub-genomic RNA4 (Martìnez et al., 2014).

To date, PMoV has only been reported in a few countries of the Mediterranean basin, in particular Italy (Caciagli et al., 1989; Parrella et al., 2021), France (Marchoux et al., 1999), Greece (Roggero et al., 2000), and Spain (Aramburu, 2001). The real incidence of PMoV was often underestimated in the past, due to the confusion generated by similar symptoms, caused by TSWV, tomato mosaic virus (ToMV), cucumber mosaic virus (CMV) with the necrogenic satellite (satRNA), and to the lack of knowledge about this virus.

**FIGURE 1:**
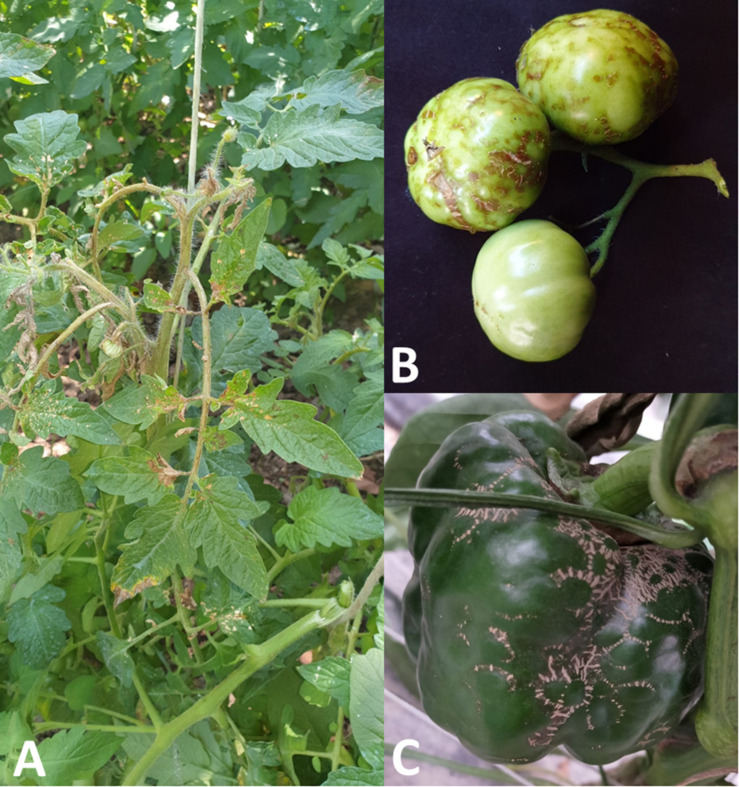
Symptoms induced by PMoV in tomato and pepper: **(A)** basal irregular necrotic lesions of tomato leaflets, longitudinal necrotic stripes of the stem and necrosis of the apex; **(B)** greenish translucent rings and corky brownish rings on deformed tomato fruits; **(C)** brownish corky rings and fruits deformations in pepper.

The symptomatology on tomato leaves consists of irregular necrotic lesions, coalescent and usually concentrated in the basal area; in addition, the necrotic lesions can perforate the leaf, which often appears deformed and curved (Figure 1A). The necrosis can progress to the leaf petiole and the stem, that often presents longitudinal necrotic stripes; in the worst cases, the stem apex necrotises and folds upon itself (Aramburu, 2001; Figure 1A). In tomato fruits the symptom consists in the pigmentation alteration: at the initial stage of infection, the fruits show greenish translucent rings, which become brownish, generally in relief, more or less corky and coalescent, causing fruit deformations (Galipienso et al., 2005; Figure 1B). On ripe and mature fruits, chlorotic rings associated to PMoV infection, have been also observed.

The symptoms on PMoV-infected pepper plants are very similar to symptoms described on tomato, consisting in corky necrotic stripes on the stems, corky rings and deformations on the fruits (Figure 1C), mosaic and basal necrosis on the leaves, causing in some cases the leaves perforation (Janssen et al., 2005).

PMoV can be passively transmitted by insects, such as thrips through the infected pollen (Aramburu et al., 2010).

Currently, the containment of PMoV dispersion can be implemented by early diagnosis and the application of preventive measures in crop management, reducing the introduction and subsequent spread in other countries. To date, there are no commercially available specific antibodies for the serological diagnosis of PMoV; the only currently available tool for PMoV identification is based on the end-point reverse transcription-polymerase chain reaction (RT-PCR) (Galipienso et al., 2008) and molecular hybridization techniques (Parrella et al., 2001, 2020; Galipienso et al., 2005). Consequently, it is necessary to develop an alternative and more sensitive diagnostic methods for the PMoV early diagnosis. For this reason, the aim of the present work was to develop a sensitive and specific molecular method to detect PMoV in infected tomato and pepper plants, based on reverse transcription-quantitative polymerase chain reaction (RT-qPCR) with TaqMan^®^ probe technique and estimate the dispersion of this pathogen in Southern Italy (Sicily).

## Materials and Methods

### Source of Viral Material

One lyophilized PMoV isolate from tomato plant, named ST-1 (GenBank Acc. No. KP234349, Galipienso et al., 2015), stored in the plant virology laboratory of the University of Palermo, was used as source material for subsequent experiments. Total RNA from 100 mg of previously re-hydrated ST-1 isolate was extracted using a Plant RNA/DNA Purification Kit (Norgen Biotek Corp., ON, Canada), following the manufacturer’s instructions. Total RNA concentration was determined in duplicate with NanoDrop 1000 spectrophotometer (Thermo Fisher Scientific, MA, United States) and used for cDNA synthesis. In the reverse transcription step (RT), the cDNA was obtained using the M-MLV Reverse Transcriptase (Thermo Fisher Scientific, MA, United States) in a 20 μL reaction volume containing 2 μL of total RNA and 100 pmol of the reverse primer PMoV-3R (5′-CACTCTTTACGTTGGCATCG-3′, position 1991–2010, Galipienso et al., 2008), according to the following cycling conditions: 42°C for 45 min and 95°C for 10 min. PCR was performed in 25 μL (final volume) containing 2 μL of the obtained cDNA, 20 mM Tris–HCl (pH 8.4), 50 mM KCl, 3 mM MgCl_2_, 0.4 mM dNTPs, 100 pmol of the forward primer PMoV-2F (5′-AATCGAGACTTTCGCCAGGA-3′, position 865–884) and 100 pmol of the reverse primer PMoV-3R (Galipienso et al., 2008), 2U of Taq DNA polymerase (Thermo Fisher Scientific, MA, United States) and RNase free water to reach the final volume, according to the following cycling conditions: 95°C for 3 min; 40 cycles of 30 s at 95°C, 45 s at 55°C, 90 s at 72°C and a final elongation of 10 min at 72°C. The PCR was carried out in a MultiGene OptiMax thermal cycler (Labnet International Inc., NJ, United States). The obtained DNA products of expected size (1,146 nt) were confirmed by electrophoretic separation in a 1.5% agarose gel stained with SybrSafe (Thermo Fisher Scientific, MA, United States).

The obtained cDNA product using the PMoV-2F/PMoV-3R primer pair was cloned in the pGEM-T vector (Promega, WI, United States) following the manufacturer’s instructions, and subsequently sequenced in both directions using an ABI PRISM 3100 DNA sequence analyzer (Applied Biosystems, CA, United States).

### Primer and TaqMan^®^ Probe Design

Multiple alignments using the ClustalX2 program (Larkin et al., 2007) were carried out using twenty PMoV CP sequences retrieved from GenBank database (Acc. No. KX866977, KP234 349, KP234348, KP234347, KP234346, KP234343, KP234342, KP 234341, KP234340, KP234339, KP234338, KP234337, KP234336, KP234335, KP234334, KP234333, KP234332, KP234331, KP2 34330, and KP234329).

Nucleotide identities between sequence pairs were estimated with the MEGA X program (Kumar et al., 2018), and two oligonucleotide primer pairs for RT-qPCR within the CP gene were designed using Vector NTI 11.5 (Thermo Fisher Scientific, MA, United States) (Table 1). Both sets of primers were tested, using the total RNA of ST-1 isolate with the concentration of ∼10 ng RNA/μL, by RT-qPCR with SYBR Green, in order to understand which primer pair has the lowest threshold cycle (Ct) value. The RT-qPCR was performed in a Rotor-Gene Q2plex HRM Platform Thermal Cycler (Qiagen, Hilden, Germany). The mixture consists in a 20 μL final volume, containing 1 μL of total RNA extract (ST-1), 1 μL of 0.5 mM of each primer, 10 μL of Master mix QuantiNova SYBR^®^ Green PCR Kit (Qiagen, Hilden, Germany) and H_2_O DEPC to reach the final volume.

**TABLE 1:** Forward, reverse primers and probe designed for RT-qPCR for the detection of PMoV.

Name	Genomic position	Referring sequence	Sequence 5′–3′	Length (nt)	Amplicon size (nt)
PMoV43 probe	1,392–1,413		FAM-CAGCGCAGGAATGCTCGCCGCG-TAMRA	22	
**PMoV21F**	1,370–1,389		CGGTGGACAAGTTTCGAACC	20	115
**PMoV115R**	1,464–1,484	KT005245	GGAAACCGGTATGACAGGTAC	21	
PMoV420F	1,769–1,788		GCACACGTACAAATGCCGAG	20	146
PMoV546R	1,895–1,914		GTCGAGAAATCGCAAACCAG	20	

*In bold the primer pair with the lowest Ct value.*

Total RNA from a healthy tomato plant and water were used as negative control samples. Each sample was analyzed twice. Cycling conditions included reverse transcription at 45°C for 10 min, incubation at 95°C for 10 min, 40 cycles of 95°C for 5 s and 60°C for 10 s; the fluorescence was measured at the end of each cycle. Melting curve steps were added at the end of RT-qPCR as following: 95°C for 1 min, 40°C for 1 min, 70°C for 1 min and a temperature increase to 95°C at 0.5°C/s to record the fluorescence.

A specific PMoV TaqMan^®^ probe (Eurofins Genomics, Luxembourg) was designed in a conserved domain within the region encompassed by the primers. The probe, with a length of 22 nucleotides, was 5′-labeled with the reporter dye FAM (6-carboxyfluorescein) and 3′-labeled with a non-fluorescent quencher (NFQ) TAMRA (Table 1). The predicted Tm values for PMoV primers and probe were 58 and 68°C, respectively, calculated with the prediction tool provided by Primer Express Software v3.0.1 (Thermo Fisher Scientific, MA, United States).

### PMoV Genotype-Specific RT-qPCR Assay With TaqMan Probe

In order to test the RT-qPCR assay with TaqMan PMoV probe, fifteen PMoV isolates from tomato and pepper plants (eight tomato and seven pepper plants), stored at the plant Virology Laboratory of the Institute for Sustainable Plant Protection of National Research Council of Italy (IPSP-CNR), Portici section (Naples), were used (Table 2). Total RNA from these samples were extracted by using a Plant RNA/DNA Purification Kit (Norgen Biotek Corp., ON, Canada), following the manufacturer’s instructions. In addition, another test was carried out with the most widespread viruses of the Mediterranean basin that affect tomato and pepper plants. In detail, the outgroup used to determine the RT-qPCR assay specificity was constituted by the following viral isolates: ToBRFV ToB-SIC01/19 isolate, ToCV (tomato chlorosis virus) Ragusa isolate, PepMV SIC2-09 isolate, CMV INU isolate, TSV (tobacco streak virus) ATCC isolate, TYLCV 8-4/2004 isolate, TYLCSV Sar-[IT:Sic:04] isolate, TICV (tomato infectious chlorosis virus) ART isolate, TSWV Turin isolate, and TMV and ToMV isolates supplied by Agdia, Inc., (Elkhart, IN, United States).

**TABLE 2:** PMoV isolates used in this study.

ID sample	Isolate name	Original Host	Geographic coordinates	Sequence acc. num.
1	Sar-1	Tomato	N39°23′48″ E8°59′27″	MN782302
2	390	Tomato	N40°37′4″E14°22′56″	MW456562
3	391A	Tomato	N40°37′4″E14°22′56″	MW456563
4	316	Tomato	N40°50′2″E14°21′5″	na
5	317	Tomato	N40°50′2″E14°21′5″	na
6	305	Tomato	N40°32′28″E14°56′26″	na
7	306	Tomato	N40°32′28″E14°56′26″	na
8	307	Tomato	N40°32′28″E14°56′26″	na
9	Fri-1	Pepper	N40°56′57″ E14°0′29″	LT160068
10	Fri-2	Pepper	N40°56′57″ E14°0′29″	LT160070
11	Pap-1	Pepper	N40°49′33″E14°21′23″	LT160069
12	419	Pepper	N41°06′30″E13°56′48″	na
13	420	Pepper	N41°06′30″E13°56′48″	na
14	421	Pepper	N41°06′30″E13°56′48″	na
15	422	Pepper	N41°06′30″E13°56′48″	na

*na, not applicable.*

RT-qPCR was performed in a Rotor-Gene Q2plex HRM Platform Thermal Cycler in a reaction mix of 25 μL final volume, containing 5 μL of total RNA extract with the concentration of ∼10 ng RNA/μL, 0.5 μM of the forward primer PMoV21F and the reverse primer PMoV115R (the primer set that yielded the most sensitive detection for all PMoV isolates providing the lowest Ct values), 0.25 μM of TaqMan PMoV43 probe, 0.5 μL of RNase Inhibitor (Applied Biosystems, CA, United States), 12.5 μL of 2X QuantiNova Probe RT-PCR Master Mix, 0.25 μL of QN Probe RT-Mix, and H_2_O DEPC water to reach final volume.

Two independent RT-qPCR assays were carried out. Each sample was analyzed in duplicate, moreover, the control samples in each test included total RNA purified from a healthy tomato plant, water instead of sample and at least two RNA transcripts (see below). The probe annealed specifically in an internal region of the PCR product amplified with primers PMoV21F and PMoV115R (primer pair that showed the lowest Ct value). The RT-qPCR conditions consisted in reverse transcription at 45°C for 10 min, enzyme denaturation at 95°C for 10 min, and 40 cycles of 95°C for 10 s and 60°C for 60 s; the fluorescence was measured at the end of each cycle. The mean (X) Ct value and the standard deviation (SD) for each sample were calculated from the four Ct values obtained.

### Standard Curve

An external standard curve was obtained in order to determine the sensitivity of the RT-qPCR protocol. Serial dilutions of an *in vitro* synthesized positive-sense RNA transcript of the selected gRNA region were amplified using the RT-qPCR TaqMan assay. The template for the *in vitro* transcription was obtained using one ST-1 clone by conventional PCR amplification with the PMoV-3R primer and a modified PMoV-2F primer with the T7 promoter (Galipienso et al., 2008).

The obtained PCR product was transcribed *in vitro* with the HiScribe^®^ T7 High Yield RNA Synthesis Kit (New England Biolabs, MA, United States) following the manufacturer’s instructions.

Transcripts were purified with RNaid Spin kit (Bio101, CA, United States), treated twice with RNase free DNase (Turbo DNA-free from Ambion, TE, United States); their concentration was determined in duplicate with NanoDrop 1000 spectrophotometer (Thermo Fisher Scientific, MA, United States).

Ten-fold serial dilutions of the transcript in healthy tomato total RNA extract (10 ng/μL) containing 10^10^–10^1^ copies were used in the RT-qPCR TaqMan assay. To ensure the absence of DNA template in transcript preparations, the assay was performed with and without the reverse transcriptase.

The RNA transcript concentration (pmol) in each dilution was calculated with the formula: micrograms of transcript RNA × (106 pg/1 μg) × (1 pmol/340 pg) × (1/number of bases of the transcript), and the number of RNA copies was calculated using this concentration value and Avogadro’s constant. The standard curve was obtained plotting the Ct values from two independent assays with four replicates per standard dilution vs. the logarithm of the RNA concentration dilution. The amplification efficiency was calculated from the slope of the corresponding curve using the formula 10^(–1/slope of the standard curve)^, or the same formula × 100 (when given as a percentage value).

### Comparison Between RT-qPCR and Conventional End-Point RT-PCR Techniques on Field Samples

A total of 130 tomato plants with and without typical symptoms induced by PMoV infection were collected in Sicily in month of June during the years 2018–2020. In detail, 40 leaf samples were collected in the 2018 (30 symptomatic and 10 asymptomatic samples), 40 leaf samples in the 2019 (30 symptomatic and 10 asymptomatic samples), and 50 samples leaf in the 2020 (35 symptomatic and 15 asymptomatic samples). The sampling areas selected were marked by GPS using the Planthology mobile application (Davino et al., 2017a). Each sample was placed in an extraction plastic bag (Bioreba, Kanton Reinach, Switzerland) containing 1 mL of extraction buffer (50 mM Tris pH 9.0, 150 mM LiCl, 5 mM EDTA), and homogenized with a HOMEX 6 homogenizer (Bioreba, Kanton Reinach, Switzerland). Healthy tomato plants used as negative controls were grown in a phytotron with a 16/8 hrs light/dark photoperiod, 27°C and 70–90% relative humidity. Total RNA from each sample was extracted by using a Plant RNA/DNA Purification Kit (Norgen Biotek Corp., ON, Canada), following the manufacturer’s instructions, and the concentration was determined in duplicate with NanoDrop 1000 spectrophotometer (Thermo Fisher Scientific, MA, United States). In order to assess the presence/absence of PMoV, the collected samples were analyzed by conventional end-point RT-PCR (Galipienso et al., 2008). Subsequently, the same samples were analyzed in duplicate by RT-qPCR, following the protocol described previously, in order to confirm the validity of the technique developed in this work and to compare the results of the two diagnostic methods.

## Results

### Source of Viral Material

The RT-PCR product of the ST-1 isolate used in this study showed the expected 1,146-bp size. The obtained sequence after cloning was trimmed in order to remove the external nucleotides, leaving only the coding region of the CP gene which showed a percentage identity of >99% with original ST-1 isolate (Galipienso et al., 2015).

### Primer and TaqMan^®^ Probe Design and Protocol Optimization

In Table 1 are reported the two primer pairs and probe designed. The PMoV21F/PMoV115R primer pair showed lower Ct value (8.64 ± 0.2) compared to the PMoV420F/PMoV546R primer pair (9.02 ± 0.2) and were selected chosen as the best candidates together with the TaqMan PMoV43 probe for the subsequent analyses (Figure not shown). The optimal annealing/extension temperature was found at 60°C and it was selected for the RT-qPCR assay.

### PMoV Genotype-Specific RT-qPCR Assay With TaqMan^®^ Probe

As reported in Table 3, the two RNA transcripts, used as positive controls, gave the most sensitive signal with a Ct value ranging from 8.09 ± 0.1 to 8.51 ± 0.2 in the four different assays. The total RNA derived from all tested PMoV isolates (15) gave also positive signal with a Ct value that ranged from 17.18 ± 0.2 to 26.86 ± 0.1, (Supplementary Figure 1), while ToBRFV, ToCV, PepMV, CMV, TSV, TYLCV, TYLCSV, TICV, TSWV, TMV, and ToMV isolates used as outgroups did not give any signal. The qRT-PCR assay confirmed its specificity since it gave no signals from healthy tomato RNA extracts and water as well. As far as the repeatability and reproducibility of the assay is considered, it was able to reliably amplify the 115-bp portion of PMoV CP gene in different experiments and laboratory conditions.

**TABLE 3:** Ct values obtained by RT-qPCR assay with TaqMan probe of fifteen PMoV isolates, two RNA transcripts, and other viruses used as outgroups.

Virus isolate	Ct ± SD
Sar-1	18.51 ± 0.2
390	24.98 ± 0.1
391A	22.22 ± 0.1
316	19.01 ± 0.3
317	18.77 ± 0.3
305	22.70 ± 0.2
306	21.89 ± 0.1
307	17.18 ± 0.2
Fri-1	21.09 ± 0.3
Fri-2	24.85 ± 0.1
Pap-1	17.69 ± 0.1
419	24.89 ± 0.3
420	18.50 ± 0.2
421	25.46 ± 0.1
422	26.86 ± 0.1
tRNA1	8.09 ± 0.1
tRNA2	8.51 ± 0.2
Healthy tomato plant tRNA	n.d.
Water	–
ToBRFV ToB-SIC01/19	n.d.
ToCV Ragusa	n.d.
PepMV SIC2-09	n.d.
CMV INU	n.d.
TSV ATCC	n.d.
TYLCV 8-4/2004	n.d.
TYLCSV Sar-[IT:Sic:04]	n.d.
TICV ART	n.d.
TSWV Turin	n.d.
TMV (Agdia, Inc.)	n.d.
ToMV (Agdia, Inc.)	n.d.

*n.d., not detected.*

### Standard Curve

**FIGURE 2:**
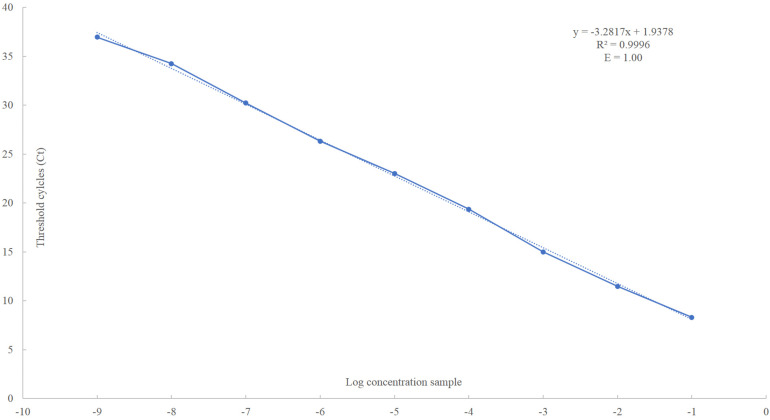
Linear regression analysis, plotting the Ct value in the Y-axis vs. the logarithm of the starting RNA dilutions in the X-axis. Each plotted point represents the mean Ct value that was calculated from the four different experiments with two replicates. The calculated correlation coefficient (*R*^2^) and amplification efficiency (*E*) values are indicated in each curve.

The obtained standard curve covered a wide dynamic range (10 units of concentration) and showed a strong linear relationship, with a correlation coefficient of 0.9996 and 100% amplification efficiency (Figure 2 and Supplementary Figure 2). The high sensitivity of the assay was achieved covering eight orders of magnitude of RNA concentration and enabling the detection of as few as 10^1^ PMoV RNA copies in tomato total RNA.

### Comparison Between RT-qPCR and Conventional End-Point RT-PCR Techniques on Field Samples

As reported in Table 4, 46 out of 130 samples resulted positive to PMoV by RT-PCR end- point, while 52 out of 130 samples give positive results to PMoV infection by RT-qPCR. In detail, the RT-qPCR showed an increase in detecting PMoV-positive samples, in fact the percentage of samples resulted positive were 40%, and 35.38% with RT-qPCR and conventional RT-PCR, respectively.

**TABLE 4:** Number of samples and percentage of positive samples analyzed by end point RT-PCR and RT-qPCR during the years 2018–2020.

Sampling year	End point RT-PCR			RT-qPCR		
	Samples analyzed	Positive samples	% Positive sample	Samples analyzed	Positive samples	% Positive sample
2018	40	9	22.50	40	9	22.50
2019	40	13	32.50	40	14	35.00
2020	50	24	48.00	50	29	58.00
Total	130	46	35.38	130	52	40.00

Regarding the difference in score between samples resulted positive at RT-PCR end-point and RT-qPCR, it is important to note that it concerns only asymptomatic samples (Table 5). Probably, this result is due to the low viral titer of asymptomatic samples. In Table 5 are reported and compared the results obtained from the two independent analyses.

**TABLE 5:** Comparison of positive field samples to RT-qPCR with end point RT-PCR results.

Sampling year	ID sample	Symptomatic	End point RT-PCR results	RT-qPCR results	Ct value 1^st^ test	Ct value 2^nd^ test
	Sic 03/18	Yes	+	+	16.15	16.23
	Sic 09/18	Yes	+	+	13.84	12.98
	Sic 10/18	Yes	+	+	20.92	20.74
	Sic 15/18	Yes	+	+	21.59	22.03
2018	Sic 19/18	Yes	+	+	19.74	20.23
	Sic 20/18	Yes	+	+	17.63	18.13
	Sic 25/18	Yes	+	+	18.62	17.15
	Sic 31/18	Yes	+	+	29.76	31.48
	Sic 37/18	Yes	+	+	19.45	18.34

	Sic 07/19	Yes	+	+	18.95	17.03
	Sic 08/19	Yes	+	+	16.89	18.12
	Sic 11/19	Yes	+	+	15.96	16.42
	Sic 15/19	Yes	+	+	14.13	13.98
	Sic 20/19	Yes	+	+	15.18	15.73
2019	**Sic 24/19**	No	−	+	29.56	29.19
	Sic 25/19	Yes	+	+	18.30	17.99
	Sic 29/19	Yes	+	+	17.65	18.25
	Sic 32/19	Yes	+	+	28.74	28.23
	Sic 33/19	Yes	+	+	16.54	16.76
	Sic 34/19	Yes	+	+	27.68	29.01
	Sic 38/19	Yes	+	+	25.78	25.81
	Sic 39/19	Yes	+	+	23.98	22.37
	Sic 40/19	Yes	+	+	19.54	19.13

	Sic 03/20	Yes	+	+	17.62	17.25
	Sic 04/20	Yes	+	+	20.33	21.27
	**Sic 06/20**	No	−	+	30.52	30.11
	Sic 07/20	No	+	+	27.89	28.46
	Sic 08/20	Yes	+	+	15.98	16.00
	Sic 10/20	Yes	+	+	15.19	14.88
	Sic 13/20	Yes	+	+	22.89	22.24
	Sic 14/20	Yes	+	+	21.64	22.78
	Sic 16/20	Yes	+	+	22.13	22.47
2020	**Sic 19/20**	No	−	+	29.33	29.98
	Sic 23/20	Yes	+	+	24.53	23.89
	Sic 24/20	Yes	+	+	23.66	23.29
	Sic 26/20	Yes	+	+	18.58	19.01
	Sic 27/20	Yes	+	+	23.47	22.96
	**Sic 28/20**	No	−	+	30.06	29.15
	Sic 30/20	Yes	+	+	16.29	16.76
	Sic 31/20	Yes	+	+	18.77	18.59
	**Sic 33/20**	No	−	+	30.75	31.09
	Sic 34/20	Yes	+	+	13.74	13.27
	Sic 35/20	Yes	+	+	19.84	19.99
	Sic 38/20	Yes	+	+	22.78	22.01
	Sic 39/20	Yes	+	+	22.59	23.08
	**Sic 40/20**	No	−	+	29.68	30.23
	Sic 43/20	Yes	+	+	14.61	15.12
	Sic 44/20	Yes	+	+	21.56	21.43
	Sic 46/20	Yes	+	+	21.15	21.36
	Sic 48/20	Yes	+	+	19.60	20.12
	Sic 49/20	Yes	+	+	22.97	23.28
	Sic 50/20	Yes	+	+	19.74	20.11

	ST-1 clone	−	+	+	11.91	12.05
	Healthy plant	−	−	−	−	−

*In bold the asymptomatic samples resulted negative to end point RT-PCR and positive to RT-qPCR.*

Regarding the PMoV spread dynamics in Sicily, during the 3 years of sampling, using the RT-qPCR assay, the incidence showed an upward trend from 22.5% in 2018 to 40% in 2020 (Table 4) in the area object of the survey, demonstrating an increase of PMoV infection of 17.5%.

## Discussion

Climate change, global trade and anthropic activities (and their consequences) seem to be closely related to the emergence of new viruses, triggering an increasing number of outbreaks and epidemics. In fact, emerging and re-emerging virus diseases represent a real threat for horticultural production, as has happened in recent years in southern Italy, with the ToLCNDV dispersion (Panno et al., 2019b) and, more recently, with the emergence and subsequent rapid ToBRFV spread in Sicily (Panno et al., 2020a) which, in the last 2 years, has been seriously compromising tomato and pepper productions.

Numerous works have highlighted that climatic conditions changes are affecting the ecological impact of some plants, including *P. officinalis*, and thus could be in some extent responsible for increasing the PMoV incidence, substantially by enlarging the flowering period, and thus the pollen production (D’Amato and Cecchi, 2008).

Pathogen detection, identification and quantification are important in plant disease control, and must be accessible in all countries to ensure eco-sustainable crop production and food safety. For these reasons, it is essential to develop, in a plant disease surveillance context, new early diagnosis methods in order to control the pathogens (Ferriol et al., 2015; Puchades et al., 2017; Panno et al., 2020c). For this purpose, distinct parameters including environment conditions, genetic characteristics, transmission methods (Ferriol et al., 2013) and agronomical practices, such as organic amendment should be considered (d’Errico et al., 2019).

Specifically, as regard virus diseases, the detection is usually performed, when available, by serological tests such as double antibody sandwich enzyme-linked immunosorbent assay (DAS-ELISA) using polyclonal or monoclonal antibodies but, in most cases, the commercial ELISA tests unavailability and/or the low viral titer in nursery plants or during the early stages of virus infection, do not allow the reliable use of these techniques (Jacobi et al., 1998).

In detail, antigen-coated plate (ACP) ELISA and direct tissue blot immunoassay (DTBIA) using a polyclonal antiserum, non-isotopic tissue-printing hybridization (TPH) and dot-blot hybridization using a specific riboprobe, and one-step or two-steps RT-PCR end-point, are the diagnostic tests developed for the specific PMoV detection by some researchers so far (Roggero et al., 2000; Parrella et al., 2001, 2016; Galipienso et al., 2005, 2015; Parrella, 2020).

Nevertheless, serological methods have some limitations because PMoV is a poorly immunogenic, producing antiserum with a low titer, even against the homologous virus (Caciagli et al., 1989) and currently no commercial kits are available for the specific serological PMoV diagnosis. In addition, TSV and prunus necrotic ringspot virus (PNRSV), which belongs, respectively, to *Ilarvirus* subgroups I and III, react with PMoV isolates, although very weakly (Lisa et al., 1998).

The one-step RT-PCR end-point has been widely applied for the diagnosis and molecular characterization of PMoV isolates (Galipienso et al., 2005, 2008). The different diagnostic methods described above shows lack of information regarding the sensitivity and specificity, therefore, there is a current need for the development of a specific and sensitive methods for PMoV identification, which could also be usefully applied to epidemiological studies.

This study describes the development and evaluation of a RT-qPCR assay for the sensitive and specific PMoV detection which, in recent years, has increased its incidence in tomato and pepper crops in southern Italy. As reported in section “Results”, the efficiency of PMoV detection in symptomatic and asymptomatic tomato samples from Sicily was higher with the RT-qPCR developed (52 out of 130 samples) compared to the conventional RT-PCR method (46 of 130 samples) described by Galipienso et al. (2008), with a sensitivity percentage increase of 4.62%. It is possible to note how the RT-qPCR assay developed in this study is more sensitive; this statement is also confirmed by the results of the sensitivity tests (see section “Standard Curve”), which showed that the developed technique is able to detect as few as 10 PMoV RNA copies in tomato total RNA.

Although it is difficult to compare the sensitivity of the PMoV RT-qPCR to the RT-qPCR assays for other ilarvirus species (Osman et al., 2014), as sensitivities are reported rarely in terms of target copy number, but in terms of sample dilution which are not directly comparable, however, a detection limit of approximately ten copies of target is consistent with assays for other, unrelated plant viruses (Agindotan et al., 2007; Gutierrez-Aguirre et al., 2009). In addition, field data demonstrate that the proposed RT-qPCR method being more sensitive, especially in asymptomatic samples, compared to those described so far for the PMoV early diagnosis, and it could represent a good and reliable tool to be applied in low viral titer conditions, during early stage of the disease, and in search for alternative hosts and latent infections.

In conclusion, the results obtained in the present study demonstrate the accuracy, specificity sensitivity, repeatability and reproducibility of the RT-qPCR assay developed for the diagnosis of PMoV in tomato and pepper plants. It is expected that the implementation of this RT-qPCR with TaqMan^®^ probe assay will facilitate efficient and fast phytosanitary certification of nursery stock, when wide samples indexing is required, thereby decreasing the risk of further spread of this dangerous virus. Moreover, the method proposed can also be useful for other application and studies, where sensitivity, accuracy and quantitative date are required, such as field survey, identification of natural virus reservoirs, and screening and characterization of resistances in germplasm.

## Data Availability Statement

The raw data supporting the conclusions of this article will be made available by the authors, without undue reservation.

## Author Contributions

SP designed and performed the experiments and bioinformatics analysis, analyzed the data, prepared figures and/or tables, authored the drafts of the manuscript, and approved the final draft. AC performed the experiments and bioinformatics analysis, analyzed the data, prepared figures and/or tables, and approved the final draft. SB performed the experiments and bioinformatics analysis, analyzed the data, and approved the final draft. SM analyzed the data, authored and reviewed drafts of the manuscript, and approved the final draft. SD conceived and designed the experiments, contributed to reagents, materials, and analysis tools, authored or reviewed drafts of the manuscript, and approved the final draft. GP conceived and designed the experiments, authored or reviewed drafts of the manuscript, and approved the final draft. All authors contributed to the article and approved the submitted version.

## Conflict of Interest

The authors declare that the research was conducted in the absence of any commercial or financial relationships that could be construed as a potential conflict of interest.

## Publisher’s Note

All claims expressed in this article are solely those of the authors and do not necessarily represent those of their affiliated organizations, or those of the publisher, the editors and the reviewers. Any product that may be evaluated in this article, or claim that may be made by its manufacturer, is not guaranteed or endorsed by the publisher.

## Supplementary Material

Supplementary Figure 1 RT-qPCR assay with TaqMan^®^ probe of fifteen characterized PMoV isolates and two RNA transcripts, using the PMoV21F/PMoV115R primer pair.

Supplementary Figure 2 Standard curves prepared with 10-fold serial dilutions of *in vitro*-synthesized RNA transcripts from PMoV ST-1 clone using RT-qPCR with TaqMan probe.
