# Supplementary figures and images for: Detection of Parietaria Mottle Virus by RT-qPCR: An Emerging Virus Native of Mediterranean Area That Undermine Tomato and Pepper Production in Southern Italy
Source: Front Plant Sci. 2021 Sep 3;12:698573. doi: 10.3389/fpls.2021.698573 (PMC8446651; doi:10.3389/fpls.2021.698573)

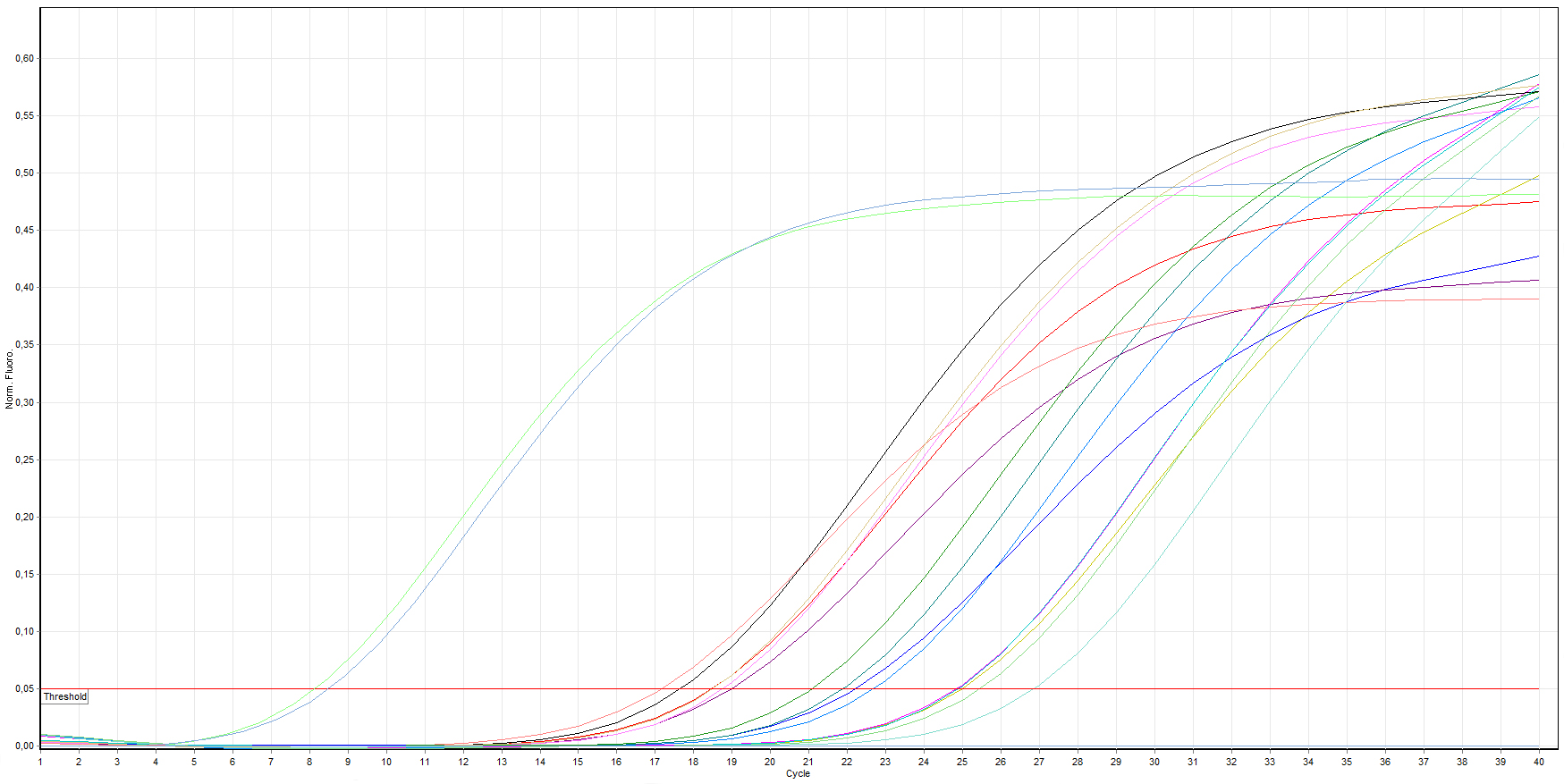

Supplement: Supplementary Figure 1 — RT-qPCR assay with TaqMan® probe of fifteen characterized PMoV isolates and two RNA transcripts, using the PMoV21F/PMoV115R primer pair. [file Image_1.JPEG]

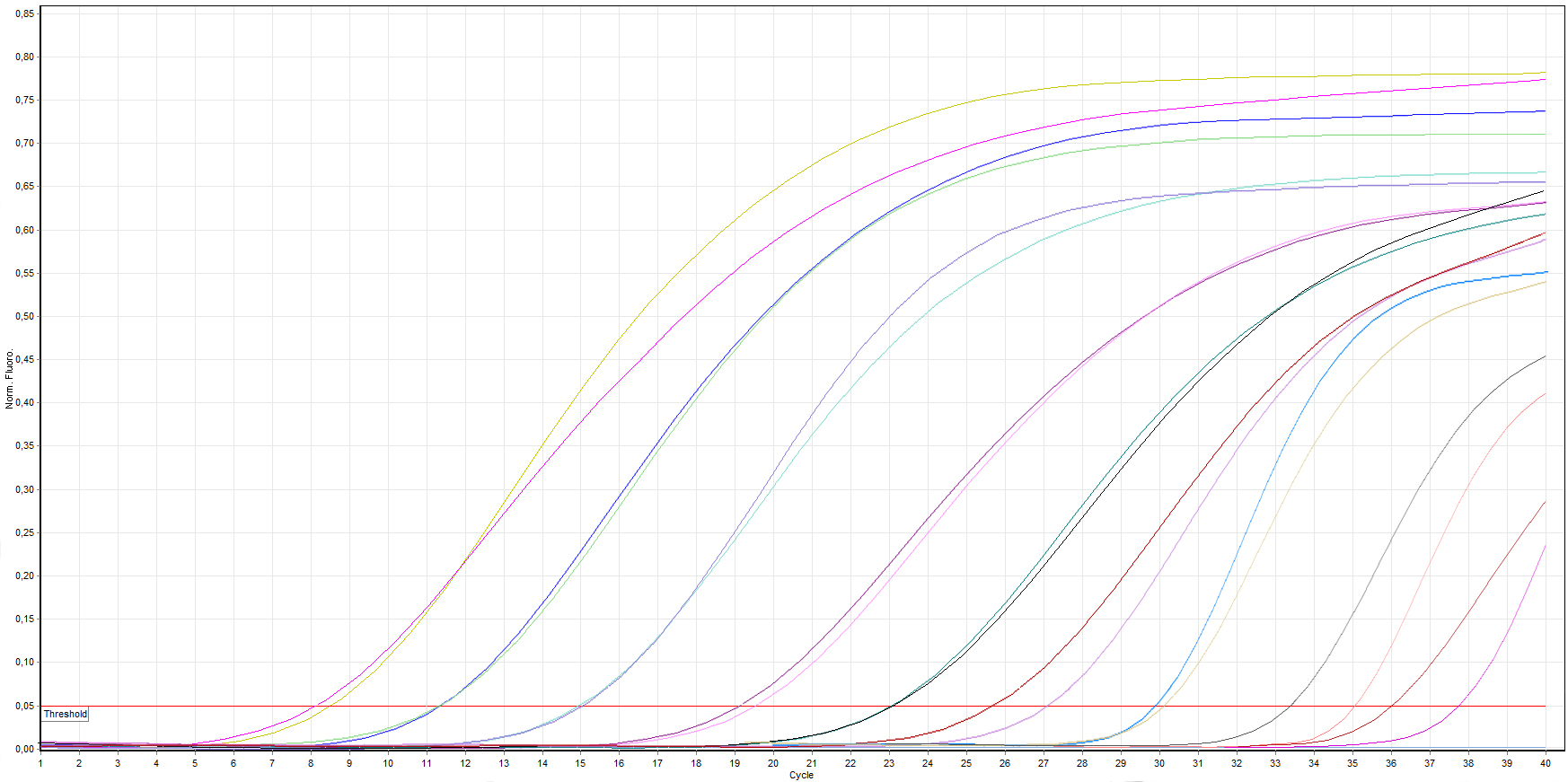

Supplement: Supplementary Figure 2 — Standard curves prepared with 10-fold serial dilutions of in vitro-synthesized RNA transcripts from PMoV ST-1 clone using RT-qPCR with TaqMan probe. [file Image_2.JPEG]
